# Synergistic Modulation of GTPase Hydrolysis and mTORC1 Activity in LRRK2-G2019S Parkinson’s Disease

**DOI:** 10.1093/geroni/igaf122.3848

**Published:** 2025-12-31

**Authors:** Zainab Cheema

**Affiliations:** “Bergen County Academies”, Maywood, New Jersey, United States

## Abstract

The LRRK2-G2019S mutation is implicated in the dysregulation of GTPase hydrolysis activity, which affects the mechanistic target of rapamycin complex 1 (mTORC1) signaling pathway in Parkinson’s disease (PD). This study aims to investigate how this mutation influences GTPase activity and its downstream effects on cellular processes in an in-vitro model of Parkinson’s Disease. It is hypothesized that treatment with Rapamycin, an mTORC1 inhibitor, in combination with Methyl 2-Hydroxybenzoate (Methyl Salicylate), a small molecule targeting GTPases, will restore GTPase hydrolysis activity, mitigate autophagic fluidity, regulate mTORC1 signaling, and reduce neurodegenerative features. Additionally, the treatment is expected to decrease reactive oxygen species (ROS) accumulation and apoptosis markers, promoting cell survival by preventing oxidative damage. Experimental techniques include MTS-based cell viability, Fluorescence-based GTPase activity, ROS-Glo, Caspase-Glo 9 and enzyme-linked immunosorbent assays (ELISA) analysis to assess changes in cell health, autophagic flux, and GTPase cycling. Genetic knockdown approaches combined with fluorescence microscopy will be used to visualize mTORC1 activity responses. Preliminary findings suggest that targeting GTPase dysfunction in mTORC1 signaling could offer novel therapeutic strategies for PD and broader neurodegenerative disease management, but further data analysis is required to validate these results.

